# Effective or just practical? An evaluation of an online postgraduate module on evidence-based medicine (EBM)

**DOI:** 10.1186/1472-6920-13-77

**Published:** 2013-05-27

**Authors:** Anke Rohwer, Taryn Young, Susan van Schalkwyk

**Affiliations:** 1 Centre for Evidence-based Health Care, Faculty of Medicine and Health Sciences, Stellenbosch University, Francie van Zijl drive, Parow 7500, Cape Town, South Africa; 2 South African Cochrane Centre, South African Medical Research Council, Tygerberg, South Africa; 3 Centre for Health Professions Education, Faculty of Medicine and Health Sciences, Stellenbosch University, Francie van Zijl drive, Parow 7500, Cape Town, South Africa

**Keywords:** Evidence-based medicine, Postgraduate, Online learning, Evaluation

## Abstract

**Background:**

Teaching the steps of evidence-based medicine (EBM) to undergraduate as well as postgraduate health care professionals is crucial for implementation of effective, beneficial health care practices and abandonment of ineffective, harmful ones. Stellenbosch University in Cape Town, South Africa, offers a 12-week, completely online module on EBM within the Family Medicine division, to medical specialists in their first year of training. The aim of this study was to formatively evaluate this module; assessing both the mode of delivery; as well as the perceived effectiveness and usefulness thereof.

**Methods:**

We used mixed methods to evaluate this module: A document review to assess whether the content of the module reflects important EBM competencies; a survey of the students to determine their experiences of the module; and semi-structured interviews with the tutors to explore their perspectives of the module. Ethics approval was obtained.

**Results:**

The document review indicated that EBM competencies were covered adequately, although critical appraisal only focused on randomised controlled trials and guidelines. Students had a positive attitude towards the module, but felt that they needed more support from the tutors. Tutors felt that students engaged actively in discussions, but experienced difficulties with understanding certain concepts of EBM. Furthermore, they felt that it was challenging explaining these via the online learning platform and saw the need to incorporate more advanced technology to better connect with the students. In their view the key to successful learning of EBM was to keep it relevant and applicable to everyday practice. Tutors also felt that an online module on EBM was advantageous, since doctors from all over the world were able to participate.

**Conclusion:**

Our study has shown that the online module on EBM was effective in increasing EBM knowledge and skills of postgraduate students and was well received by both students and tutors. Students and tutors experienced generic challenges that accompany any educational intervention of EBM (e.g. understanding difficult concepts), but in addition had to deal with challenges unique to the online learning environment. Teachers of EBM should acknowledge these so as to enhance and successfully implement EBM teaching and learning for all students.

## Background

Evidence-based medicine (EBM) is no longer a new term - it has been around for twenty years. This paradigm was introduced in the seminal JAMA article in 1992 [1] and has since been described as “*one of the 15 greatest medical milestones since 1840*” by the British Medical Journal [2]. The widely used definition of EBM by David Sackett (1996) explains the concept well. It is “*the conscientious, explicit and judicious use of the current best evidence in making decisions about the care of individual patients”*[3], bringing together external evidence that informs about the effects of new tests, treatments and interventions; clinical judgement and expertise of the clinician; and the patient’s clinical state, values, preferences, needs and predicament. The process of EBM typically includes five steps: Formulating an answerable question from a clinical problem; finding the best available evidence applicable to the problem; critically appraising the evidence for validity, clinical relevance and applicability; interpreting and applying the results of the evidence in the clinical setting; and evaluating the performance [4].

It can be argued that teaching the steps of EBM to undergraduate as well as postgraduate health care professionals is crucial for the implementation of effective, beneficial health care practices and the abandonment of ineffective, harmful ones. Health care professionals need to learn how to deal with the challenge of information overload. Paul Glasziou suggested: “*The search engine is now as essential as the stethoscope*” [5]. The importance of the knowledge, skills and attitudes learnt through the principles of EBM was also highlighted in the recent Lancet report on the health professional for the 21^st^ century [6], which proposed that health care professional training should become transformative. Transformative learning aims to develop change agents – graduates with leadership attributes, who can function in a team within the local health systems. One of the fundamental shifts inherent in transformative learning is closely aligned to the steps of EBM - the shift from memorization of facts to *“critical reasoning that can guide the capacity to search, analyse, assess and synthesise information for decision-making”*[6]*.*

A number of systematic reviews, looking at the effects of teaching EBM to both under- and postgraduate health care professionals, have been conducted. These include teaching EBM in the form of journal club meetings as well as classroom based teaching [7-12]. Even though the quality of these reviews (as well as of the numerous included studies) varies, results show increased EBM knowledge and skills directly after a teaching event. Coomarasamy and Khan [7] concluded that integrated teaching of EBM, compared to stand-alone teaching of EBM, not only increases knowledge and skills, but also influences EBM attitudes and behaviour. Subsequently they have proposed a hierarchy for EBM teaching and learning [13] where adopting an interactive and clinically integrated approach is the most effective way of learning EBM.

A modern-day feature of education programmes, including those for health care professionals, is that of online learning. Although some of the systematic reviews mentioned above referred to studies of online learning modules, none of the reviews have specifically addressed online learning of EBM. A cluster randomized controlled trial done in the United Kingdom, found that an online module on EBM was just as effective as a face-to-face lecture based module in increasing knowledge of graduate doctors [14]. A systematic review on internet-based learning has shown that online learning in the medical field is neither superior nor inferior to face-to-face learning [15]. But, as Khan and colleagues have noted, the variations among instructional methods and the rapid advancement of technology make it difficult to determine which elements contribute to an effective online learning environment for EBM specifically [13].

When considering the attributes of online learning in postgraduate medical education, one can argue that these fit well within the EBM paradigm. Online learning enables adult learners to tailor their learning according to their unique needs (open learning), giving them autonomy over their learning and increasing intrinsic motivation. Enhanced learning occurs through internal motivation, rather than external drivers, and requires acknowledgement of shortcomings and adoption of a reflective approach towards one’s own practice [16]. The attributes of accessibility and convenience (distributed learning) personalise the learning, because students decide when and where they are receptive to learning [17-19]. The alignment between attributes of online learning and EBM foci suggests that making use of online learning in this context could be of value.

Although there are many advantages of online learning, one also needs to consider challenges and disadvantages thereof. These include social isolation, de-individualised instruction, cost, technical problems and poor instructional design [20,21].

Stellenbosch University offers an online EBM module, with no contact sessions, within the Family Medicine Division, to medical specialists in the first of their four years of training. This module was developed in 1997, originally covering formulation of questions, searching the literature and critical appraisal; and was offered in a face-to-face mode, with weekly lectures over the period of a year, to an average of 15 Family Medicine specialists in training. In 2003, when the number of students from outside of Cape Town increased, the Family Medicine Division decided to transform the face-to-face module to a fully online module, offered over 12 weeks. This also covered formulating questions from clinical practice, searching the literature for answers to the questions, critically appraising the literature and using the results when making decisions about individual patient care. The initial online module ran from 2004 to 2008 and included a summative assessment task, as well as weekly interactive chat room sessions. In 2008, the module was adapted. The summative assessment task was extended to include formative, individual assessments, and group work. The chat room sessions were abandoned and replaced by a discussion forum on the learning management system. The module was structured into themes relating to the steps of EBM (see Table 1).

**Table 1:** Structure of the EBM module

Week 1-2	Introduction to EBM and phrasing questions
Week 3-4	Searching the literature
Week 5-6	Introduction to critical appraisal, the GATE framework and study design
Week 7-10	Applying the GATE framework and other critical appraisal guidelines to studies
Week 11	Considering application of evidence in practice
Week 12	Final assignment

Since 2009, the module has been running in its current format and is being offered to an average of 50 students annually, the majority of whom are based outside of Cape Town and across Africa.

We wanted to know what we could learn from an existing online EBM module, with a view to developing similar modules in future. We therefore endeavoured to formatively evaluate this module by answering the following questions: Does the content of the module foreground the steps involved in EBM? How do the students experience learning? What are the tutors’ perspectives on the content and organization of the module? The purpose of this study was therefore to assess the mode of delivery, as well as the perceived effectiveness and usefulness of the EBM module.

Formative evaluation, as opposed to summative evaluation, is conducted during the course of a programme, and aims to improve or adapt materials, methods, activities and organization of the programme concerned [22]. In this article we present the key findings of this evaluation, focussing on the challenges experienced by both students and tutors in our study.

## Methods

**Figure 1:**
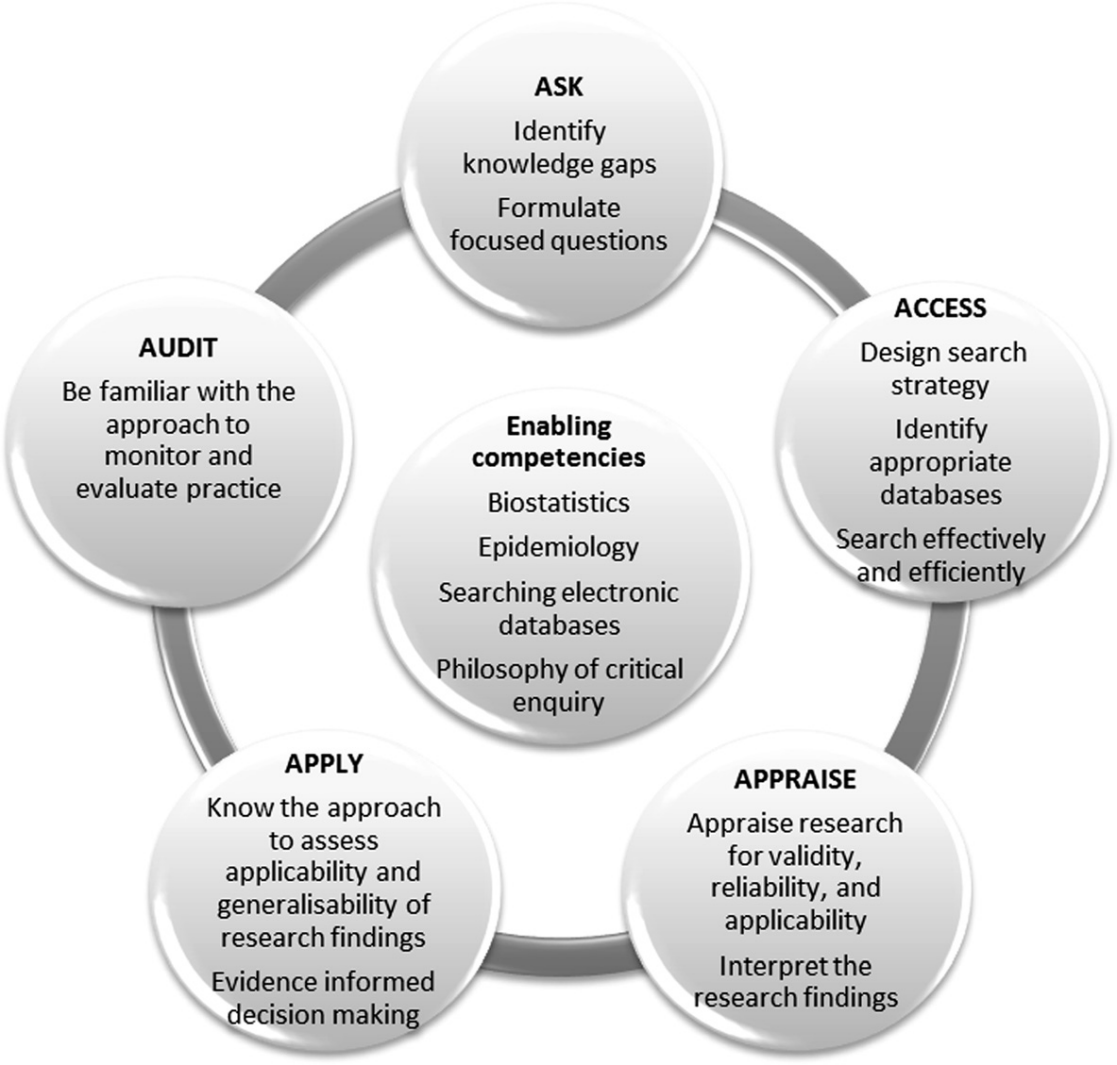
EBM key and enabling competencies.

We identified essential EBM competencies, drawing on the CanMeds model [23], which contains within one of its domains (scholar), enabling competencies that resonated with our understanding of the steps of EBM. This was further informed by a literature review and discussion with faculty members as well as experts in the field. The competencies include five key competencies that mirror the five steps of EBM, as well as certain enabling competencies - basic concepts like epidemiology and biostatistics, that students need to understand before they can practice EBM (Figure 1). In our view, a comprehensive module on EBM should address both the key and the enabling competencies. Ethical approval was obtained from the ethics committee at Stellenbosch University (N11/09/303).

We used mixed methods for the evaluation. Firstly, we conducted a document review, describing the overall structure and the progression of the course; and compared learning outcomes with the pre-specified EBM competencies, in order to assess the extent of EBM coverage. We evaluated learning material, methods and assessments to determine the extent of their alignment with the EBM competencies.

Secondly, we invited tutors involved in the module to participate in individual, semi-structured interviews. Their perspectives on the module, including the benefits, barriers and challenges related to an online module were explored using open-ended questions. We obtained informed consent before the interviews were conducted, recorded, transcribed and coded them all manually. Although initial coding was undertaken by the lead author (AR) who identified emerging themes according to the pre-specified questions, these were critically reviewed by the other authors (TY and SvS) and discussed relative to the other data sets prior to finalising the discussion section of the article.

Lastly, after completion of the module, we asked students to complete an anonymous, electronic questionnaire. We adapted questions from Baum et al. [24] to determine their attitudes towards EBM; and asked them to rate their general experiences of the module as well as the usability and user friendliness of the e-learning platform using a five-point Likert scale. The following example is illustrative of the type of questions students were asked to answer:

•Please rate the following: EBM is realistic to practice in routine patient care 1: strongly disagree; 2: disagree; 3: don’t know; 4: agree; 5: strongly agree

The last part of the questionnaire contained questions to determine self-perceived confidence in EBM using a confidence scale of 0% to 100%, adapted from the evidence-based practice confidence (EPIC) scale [25]. The following example is illustrative of the type of questions students were asked to answer:

•*Please rate your confidence in your ability to:* Develop a search strategy based on the question

•0% 10% 20% 30% 40% 50% 60% 70% 80% 90% 100%

•(*0% = no confidence; 100% = completely confident)*

Scott (2008:19) suggests that a curriculum comprises four dimensions: “aims or objectives, content or subject matter, methods or procedures, and evaluation or assessment” [26]. We used these variables to evaluate the module according to the five key competencies of EBM.

## Results

### Document review

The document review indicated that the EBM module covered most EBM competencies: phrasing answerable questions; accessing the literature; critical appraisal of the literature and the application of research results in the clinical practice. Although learning outcomes were not always explicitly stated, the content of the module comprehensively addressed important EBM aspects. The key focus was, however, on questions about effects of interventions, while other types of questions (e.g. risk factor or diagnostic questions) were only briefly covered. Learning was facilitated by the provision of reading material, online examples, self-tests, practicing of the different EBM steps and discussions with peers and tutors on the online platform. All examples discussed amongst the students, were derived from scenarios that they themselves experienced in their clinical practice. Students were assessed separately for each competency as well as for their contributions to the discussions. The final assignment required them to go through the whole EBM process based on a knowledge gap they encountered in their own practice. The summary of this evaluation according to the five EBM key competencies is presented in Table 2.

**Table 2:** Summary of the module evaluation related to the key EBM competencies

**Competency**	**Ask**	**Access**	**Appraise**	**Apply**	**Audit**
**Learning outcomes aligned with competency?**	Yes	No learning outcomes	Yes	No learning outcomes	Not addressed
**Curriculum content aligned with competency?**	Yes	Yes	Yes	Yes	N/A
**Curriculum material adequate**^**1**^**?**	Yes	No	No	No	N/A
**Teaching and learning methods**	Reading material	Reading material	Reading material	Reading material	N/A
	Examples	Examples	Examples		
	Practicing formulation of questions with peers	Demonstration of searches	Self-tests		
		Practicing searching	Discussions with peers and tutor		
	Discussions with peers and tutor	Discussions with peers and tutor			
**Assessment aligned with learning outcomes?**	Yes	No learning outcomes	Yes (Not all learning outcomes assessed)	No learning outcomes	N/A
**Level of assessment**^**2 **^**[**[27,28]**]**	Knows how	Knows how	Knows how	Knows how	N/A
**Students’ competency**	Class average: 62%	Class average: 62%	Class average: 87%	Final assignment average: 60%	N/A
**Students’ self-perceived confidence**^**3 **^**[**[25]**]**	Range of scores:	Range of scores:	Range of scores:	Range of scores:	Range of scores:
	60-100% (average 85%)	30-100% (average 82%)	40-100% (average 77%)	50-100% (average 84%)	50-100% (average 82%)
**Students’ attitude**^**4 **^**[**[24]**]**	52% positive	46% positive	92% positive	87% positive	N/A
	48% negative	56% negative	8% negative	13% negative	

^1^Judgements about adequacy of material related to content on EBM enabling and key competencies, which we compared to the material used in the EBM module.

^2^According to Miller’s pyramid of competence [27,28].

^3^According to EPIC scale that measures self-perceived EBM competence on a scale from 0% (no confidence) to 100% (completely confident).

^4^Based on Likert score ratings of EBHC statements adapted from Baum (2003), from strongly disagree to strongly agree.

### Students’ experience of the module

Of the 43 students registered for the EBM module in 2011, 24 (56%) responded to the electronic survey. Students were based all around South Africa and in other African countries (e.g. Namibia, Botswana, Zimbabwe and Uganda). Although we did not ask students to comment on previous exposure to EBM teaching, we know that there is a lack of formal EBM teaching at an undergraduate level at Stellenbosch University (Rohwer A, Young T, et al.: Document review of undergraduate MB,ChB curriculum to inform enhancement of public health (PH), evidence-based health care (EBHC), health systems and services research (HSSR) and infection prevention and control (IPC) training at undergraduate level*, unpublished report*; Rohwer A, Young T, et al.: The young doctor’s opinion on Evidence-based Health Care (EBHC) in Stellenbosch University’s medical curriculum, *unpublished report)*. This was also confirmed by the tutors, who explained that most students were being exposed to EBM teaching for the first time during this module.

As mentioned above, students used scenarios from their own clinical practice to practice formulating answerable questions and finding relevant studies. Students’ rating of their own attitude towards EBM in the questionnaires revealed that more than half of them found literature searches too time consuming to do in a clinic. In addition the survey showed that they were not very confident in their ability to perform literature searches.

The bulk of the module focused on critical appraisal. Students were required to do group work that focused on critical appraisal of randomized controlled trials and clinical guidelines. They reported being least confident in their ability to critically appraise strengths and weaknesses of different study designs, determine the validity of a study and interpret the results with ease. They also felt that they needed more information on statistical concepts. Nonetheless, the questionnaire showed that they appreciated the value of different types of studies. Most students felt that EBM was relevant to their practice.

Overall, students were satisfied with the delivery of the module on the online platform although it transpired that they would have liked more input from the tutors. Some students felt that participation of not only tutors, but fellow students in the online discussions was lacking and others mentioned that they simply disliked online learning. It was evident, however, that international students greatly appreciated the opportunity to enrol in the module and interact with colleagues all over the world.

### Tutors’ perceptions of the module

According to the tutors, students engaged actively in discussions on the online platform about the questions they were planning to explore.

*“In the first week, there probably were about 300 different posts related to all the questions. So they really engaged and critiqued and commented on each other’s questions. And once they exhausted the question, then somebody else would post a scenario”*.

There was, on the other hand, also a feeling that the students found the new terminology as well as the articulation of a specific problem daunting at first.

*“You know what the problem is, but how do you articulate the problem? I found that a lot of the learners have struggled with just articulating the problem. Something as simple as a focused, answerable question … they struggle with that”*.

Tutors picked up that not all students were comfortable with the process of searching electronic databases to find the best available evidence.

*“I think that the search processes are also a little bit challenging and the Boolean terms and just…understanding what tools they can use to get the articles or the information they require. I noticed that there is a little bit of anxiety that is related to that”*.

Tutors were aware of this perceived barrier to using EBM in practice, but felt that it was unfounded.

“They always use the excuse that there is not enough time and so on. But we do try to tell them that using it in your own practice, so identifying problems that you experience, not only using it for the sake of using it”.

Tutors also argued there was room for improvement in the teaching of critical appraisal, echoing the need of the students for more information on certain statistical and critical appraisal aspects. There was disparity among the tutors on the tools (such as the GATE framework and User’s Guide to Critical Appraisal) used for teaching critical appraisal. While some tutors found them very useful and user-friendly, others found that they did not provide sufficient support for critical appraisal at a Master’s level.

*“Should a doctor be an epidemiologist, or should he just implement the guideline when it comes to (him), or should he critically reflect? (…) How much should they know? We settled for what they currently have in their module. Bearing in mind that there is room for improvement”*.

Critical appraisal and a doctor’s comprehension thereof, was seen as being essential for quality reading and implementation of the evidence in practice, but also the most difficult aspect of EBM to teach to students.

Tutors mentioned the benefits of having an online module, which included the ability of students from around Africa to participate in the module; the cost-effectiveness thereof; students being able to work at their own time and their own pace *“because, our students really cannot afford being away from their practices”*; being able to learn while practicing; and being less inhibited to participate in discussions and ask questions.

“Some might feel more at ease to ask questions, or to maybe express themselves, whereas in the group in the class they…maybe feel more inhibited”

Disadvantages of having a completely online module were also highlighted. The main challenge that emerged for the tutors was explaining difficult concepts like critical appraisal and practical skills like searching techniques via emails and online discussions, without the ability of drawing diagrams, demonstrations and immediate responses to questions.

*“There are, however, some things, if you think of some of those critical appraisal aspects, is sometimes better explained in a contact session, compared to having it online”*.

These perspectives, however, could be challenged as there are new technologies available that would make such enhanced interactivity plausible.

Indeed, tutors across the board felt that there was room to improve the online environment with new technologies that could support learning.

“I think it is important that the tools we are using support the module”.

Amongst those mentioned were chat rooms, blogs, video- and multimedia clips, screencasts, RSS feeds of activity on the learning management system, as well as LinkedIn, Facebook or Twitter accounts that could be used to stay connected beyond the completion of the module.

“I think the chatroom gives you the opportunity to ask in real time and to challenge yourself and your tutor and your colleagues, you know, as things unfold”. “There are blogs and there are social media sites (sic) and there are several avenues you can explore. LinkedIn for example, you create a group on LinkedIn and you have constant feedback and you have an opportunity for you to give them new information about EBM as well”

Tutors also felt strongly about the importance of making EBM applicable and relevant to students’ clinical practice.

*“If it is just pure theoretical and academic discussion and it is not going to translate into changing your practice, you are missing the point. So they are enjoying and they see the relevance clearer, when you have it linked to a case”*.

Tutors emphasised that students should understand that the questions arise from problems they experience in their clinical field.

*“It’s a problem that stems from patient interaction and that is where you sort of initiate the process of asking a question. So in the end, I think they do get it and they can see it that they can use it in their busy practice”*.

Without practical application and integration of learning, it becomes *“like teaching somebody to swim from a textbook or riding a bike from a manual”.*

## Discussion

We evaluated the content as well as the learning platform of an online EBM module offered to Family Medicine specialists in training at Stellenbosch University. The twelve week module offers postgraduate students the opportunity to integrate EBM learning into their daily practice. The module covers four of the five key competencies of EBM and takes the students through the process of EBM, requiring them to think about knowledge gaps encountered in their own practice.

Students’ self-perceived confidence in EBM knowledge and skills showed that they were least confident in their ability to critically appraise a study on effects of interventions, even though the bulk of the module focused on this aspect of EBM. This indicates that there is a need to improve teaching of critical appraisal skills within this module, although this is an example of a generic challenge of EBM learning – it is not unique to the online environment. Future online offerings will need to take cognisance of this reality and intentionally seek to focus on opportunities that will directly enhance these skills.

Generic challenges included the difficulties students experienced with regards to the new terminology; aspects of finding the literature; as well as understanding statistical concepts and critical appraisal. On the other hand, challenges experienced by the tutors related to choosing the best study material and making learning as relevant and applicable as possible. Despite the numerous publications on teaching and learning of EBM, the challenges remain. There is a need for additional studies on overcoming perceived barriers to learning and practicing EBM if we are going to foster a cohort of health practitioners who can effectively adopt EBM as part of their daily practice.

This is linked to the perception among students that EBM is too time-consuming for them to apply in their every-day practice. A recent systematic review on the barriers to general practitioners’ use of EBM [29] showed that a lack of EBM skills; a lack of a positive attitude; and a lack of positive attitudes of colleagues, were common barriers. Other barriers included a lack of time and a lack of access to information. Ironically, information overload (amount of published articles) was listed as a barrier, when in fact we would argue that it should be motivating health care professionals to acquire the skills to deal with this overload.

Even though a prerequisite for practicing EBM is the acquisition of specific knowledge and skills, it also requires a shift in attitude; role models in the clinical field; resources to support evidence-informed practices; and a commitment to life-long learning. The question whether these requirements can be met by online learning remains – bringing us to the challenges that are unique to teaching EBM on an online platform, one of them being the difficulty of creating an authentic learning environment.

Authentic learning designs should provide authentic contexts and tasks as well as access to expert input and multiple perspectives. They should support collaborative construction of knowledge; promote reflection and articulation; provide coaching and scaffolding by the teacher at critical times as well as authentic assessment of learning [30]. A recent systematic review summarising ways to improve internet-based learning concluded that improved learning is associated with interactivity, practice exercises, repetition and feedback [31].

Our study shows that the EBM module ticks most of these boxes: It requires students to take real examples from their clinical practice and discuss these with their peers and tutors to construct new knowledge. It encourages interaction, reflection and articulation of new terminology – yet, it does not seem to fulfil all the expectations of the students and the tutors. There appears to be a need for further improving connectedness between students and tutors and sensitising them to the challenges, while also highlighting the potential of this mode of teaching to enhance aspects of the learning experience. Using social media (Facebook, blogs, Twitter, Wikis) in a formal learning setting, for example, has been shown to promote the development of informal and formal learning communities, where students feel that they are a part of a classroom in which their opinions matter [32]. It also affords them the opportunity to manage their own learning spaces and connections with fellow students outside of the formal context. This can be compared to informal discussions in a physical classroom [32]. Incorporating social media in this module thus has the potential to enhance learning and both learner and tutor satisfaction with learning.

One of the limitations of our study is that it evaluated self-perceived EBM confidence and attitudes directly after the module. It would be worthwhile repeating these assessments after a year or two to get an idea of the longer term impact on clinical decision-making. We found no other study looking at long term effects of EBM teaching on EBM practice in the clinical field. Qualitative studies on students’ perspectives on EBM could also provide more in depth information about the application of EBM in practice.

Even though we have mostly addressed the challenges that an online learning environment provides, there are also advantages that were pointed out by both tutors and students. The most obvious advantage was the availability of the module to students all around South Africa and the rest of Africa. This has crucial implications for the enhancement of health care on the continent. The pedagogical principles of online learning allow tailoring of learning according to individual learning needs. Online learning also encourages participants from across the globe to share experiences and construct new knowledge as a community.

## Conclusion

Our evaluation has shown that the online EBM module is effective in increasing EBM knowledge and skills of postgraduate students and was well received by both students and lecturers, but that it does not overcome the documented generic challenges experienced when teaching EBM in any format. Advantages of an online module include the ability to interact with a global network of colleagues; and the opportunity to tailor one’s learning according to individual needs, as well as time and space preferences.

However, as with any teaching, presenting an online module comes with unique challenges, some of which could be addressed by incorporating emerging technologies to create a more authentic learning environment. With the rapid advancement of technology, it is inevitable that online learning will continue to play a vital role in the education of health care professionals. It is imperative that we use these features to our advantage and continue working on optimising online learning of EBM if we want to see the impact on health outcomes.

Being cognisant of and responsive to these challenges could help teachers of EBM to design and implement EBM teaching more successfully.

## Abbreviations

EBM: Evidence-based medicine; EPIC: Evidence-based practice confidence; GATE: Graphic approach to epidemiology.

## Competing interests

The authors declare that they have no competing interests.

## Authors’ contributions

AR, TY and SvS contributed to the conceptualisation of this study. AR led the data collection and all authors contributed to data analysis and interpretation. AR wrote the manuscript with input from SvS and TY. All authors read and approved the final manuscript.

## Authors’ information

AR is a researcher at the Centre for Evidence-based Health Care, Stellenbosch University, South Africa, with a background in Clinical Epidemiology. In addition to doing research, she teaches EBM to undergraduate and postgraduate students, as well as health care professionals in the field. She has a special interest in online learning.

TY is the Director of the Centre for Evidence-based Health Care at Stellenbosch University, South Africa, and consultant to the South African Cochrane Centre. She is an epidemiologist with a specialist degree in public health and considerable expertise in the field of evidence‒based health care. She has coordinated international collaborative projects which facilitate the use of best evidence in health care policy and practice, conducted many systematic reviews and provided intensive training, mentorship and editorial support to authors of Cochrane and other systematic reviews. Integral to her work is the co‒ordination and evaluation of training programmes for under‒ and postgraduate students in epidemiology, evidence‒based health care and research synthesis nationally and in the African region.

SvS is Deputy-Director (Education) at the Centre for Health Professions Education, Faculty of Medicine and Health Sciences, Stellenbosch University, South Africa. In addition to teaching on different Masters’ programmes and supervising PhD students, she is involved in numerous staff development initiatives in the Faculty. SvS is the educational advisor on the Stellenbosch University Rural Medical Education Partnership Initiative (SURMEPI) and research interests include medical education, doctoral supervision and rural health.

## Pre-publication history

The pre-publication history for this paper can be accessed here:

http://www.biomedcentral.com/1472-6920/13/77/prepub

## References

[B1] group E-bmw Evidence-based medicine - a new approach to teaching the practice of medicine JAMA 1992 268 17 2420 2425 10.1001/jama.1992.03490170092032 1404801

[B2] Montori VM Guyatt GH Progress in evidence-based medicine JAMA 2008 300 15 1814 1816 10.1001/jama.300.15.1814 18854545

[B3] Sackett DL Rosenberg WM Gray JAM Haynes RB Richardson WS Evidence-based medicine: what it is and what it isn’t Br Med J 1996 312 71 10.1136/bmj.312.7023.71 8555924 PMC2349778

[B4] Dawes M Summerskill W Glasziou P Cartabellotta A Martin J Hopayian K Porzsolt F Burls A Osborne J Sicily statement on evidence-based practice BMC Med Educ 2005 5 1 1 10.1186/1472-6920-5-1 15634359 PMC544887

[B5] Glasziou P Burls A Gilbert R Evidence-based medicine and the medical curriculum - the search engine is now as essential as the stethoscope BMJ 2008 337 704 705 10.1136/bmj.a704 18815165

[B6] Frenk J Chen L Bhutta Z Cohen J Crisp N Evans T Fineberg H Garcia P Ke Y Kelley P Health professionals for a new century: transforming education to strengthen health systems in an interdependent world Lancet 2010 376 1923 1958 10.1016/S0140-6736(10)61854-5 21112623

[B7] Coomarasamy A Khan KS What is the evidence that postgraduate teaching in evidence based medicine changes anything? A systematic review BMJ 2004 329 1017 10.1136/bmj.329.7473.1017 15514348 PMC524555

[B8] Ebbert JO Montori VM Schultz HJ The journal club in postgraduate medical education: a systematic review Med Teach 2001 23 5 455 461 12098365 10.1080/01421590120075670

[B9] Taylor R Reeves B Ewings P Binns S Keast J Mears R A systematic review of the effectiveness of critical appraisal skills training for clinicians Med Educ 2000 34 120 125 10.1046/j.1365-2923.2000.00574.x 10652064

[B10] Horsley T Hyde C Santesso N Parkes J Milne R Stewart R Teaching critical appraisal skills in healthcare settings (Review) The Cochrane Library 2011 11 Art. No.: CD001270 10.1002/14651858.CD001270.pub2 PMC7389530 22071800

[B11] Alguire PC A review of Journal Clubs in postgraduate medical education J Gen Intern Med 1998 13 347 353 10.1046/j.1525-1497.1998.00102.x 9613892 PMC1496950

[B12] Audet N Gagnon R Ladouceur R Marcil M L’enseignement de l’analyse critique des publications scientifiques medicales est-il efficace? Revision des etudes et de leur qualite methodologique Can Med Assoc J 1993 148 6 945 952 8448709 PMC1490748

[B13] Khan KS Coomarasamy A A hierarchy of effective teaching and learning to acquire competence in evidenced-based medicine BMC Med Educ 2006 6 59 10.1186/1472-6920-6-59 17173690 PMC1770917

[B14] Hadley J Kulier R Zamora J Coppus SF Weinbrenner S Meyerrose B Decsi T Horvath AR Nagy E Emparanza JI Effectiveness of an e-learning course in evidence-based medicine for foundation (internship) training J R Soc Med 2010 103 7 288 294 10.1258/jrsm.2010.100036 20522698 PMC2895523

[B15] Cook DL Garside AJ Dupras S Erwin DM PJ Montori VM Internet-based learning in the health professions: a meta-analysis JAMA 2008 300 10 1181 1196 10.1001/jama.300.10.1181 18780847

[B16] Race P Making learning happen: a guide for postcompulsory education 2005 London: Sage

[B17] Ruiz JM MJ Leipzig RM The impact on e-learning in medical education Acad Med 2006 81 3 207 212 10.1097/00001888-200603000-00002 16501260

[B18] Dabbagh N Pedagogical models for e-learning: a theory-based design framework Int J Technol Teach Learn 2005 1 1 25 44

[B19] Clark D Psychological myths in e-learning Med Teach 2002 24 6 589 604 10.1080/0142159021000063916 12623452

[B20] Cook D Web-based learning: pros, cons and controversies Clin Med 2007 7 1 37 42 10.7861/clinmedicine.7-1-37 17348573 PMC4953546

[B21] Spice R Palacios M Biondo PD Hagen NA Design and implementation of an online course on research methods in palliative care: lessons learned J Palliat Med 2011 14 4 413 419 10.1089/jpm.2010.0374 21375396

[B22] Goldie J AMEE Education Guide no. 29: evaluating educational programmes Med Teach 2006 28 3 210 224 10.1080/01421590500271282 16753718

[B23] Frank J The CanMEDS 2005 physician competency framework. Better standards. Better physicians. Better care 2005 Ottawa: The Royal College of Physicians and Surgeons of Canada

[B24] Baum KD The impact of an evidence-based medicine workshop on residents’ attitudes towards and self-reported ability in evidence-based practice Med Educ Online 2003 8 4 10.3402/meo.v8i.4329 28253169

[B25] Salbach NM Jaglal SB Creation and validation of the evidence-based practice confidence scale for health care professionals J Eval Clin Pract 2011 17 4 794 800 10.1111/j.1365-2753.2010.01478.x 20630014

[B26] Scott D Critical essays on major curriculum theorists 2008 London: Routledge

[B27] Miller GE The assessment of clinical skills/competence/performance Acad Med 1990 65 9 563 567

[B28] Waas V Van der Vleuten C Shatzer J Jones R Assessment of clinical competence Lancet 2001 357 945 949 10.1016/S0140-6736(00)04221-5 11289364

[B29] Zwolsman S te Pas E Hooft L Wieringa-de Waard M van Dijk N Barriers to GPs’ use of evidence-based medicine: a systematic review Br J Gen Pract 2012 62 600 e511 e521 10.3399/bjgp12X652382 22781999 PMC3381277

[B30] Herrington J Reeves TC Oliver R A guide to authentic e-learning 2010 New York: Routledge

[B31] Cook DA Levinson AJ Garside S Dupras DM Erwin PJ Montori VM Instructional design variations in internet-based learning for health professions education: a systematic review and meta-analysis Acad Med 2010 85 909 922 10.1097/ACM.0b013e3181d6c319 20520049

[B32] Dabbagh N Kitsantas A Personal learning environments, social media, and self-regulated learning: a natural formula for connecting formal and informal learning The Internet Higher Educ 2012 15 1 3 8 10.1016/j.iheduc.2011.06.002

